# Regional differences in energy allocation of black sea bass ( *Centropristis striata* ) along the U.S. Northeast Shelf (36°N to 42°N) and throughout the spawning season

**DOI:** 10.1111/jfb.15023

**Published:** 2022-03-09

**Authors:** Emily Slesinger, Kiernan Bates, Mark Wuenschel, Grace K. Saba

**Affiliations:** ^1^ Department of Marine and Coastal Sciences Rutgers University New Brunswick New Jersey USA; ^2^ Alaska Fisheries Science Center, National Marine Fisheries Service National Oceanic and Atmospheric Administration Newport Oregon USA; ^3^ Northeast Fisheries Science Center, National Marine Fisheries Service National Oceanic and Atmospheric Administration Woods Hole Massachusetts USA

**Keywords:** black sea bassenergy allocationfish reproductionlipid contentU.S. Northeast Shelf

## Abstract

Fish reproduction is energetically costly, leading to a suite of energy allocation strategies for maximizing lifetime reproductive potential. Assessing energetic allocation for species that inhabit a wide distributional range can provide insight into different strategies found across individuals and populations. The Northern stock of black sea bass (*Centropristis striata*) inhabits the U.S. Northeast continental shelf from Cape Hatteras, NC, to the Gulf of Maine, and spawns inshore throughout this distribution from April to October. To assess energy allocation towards spawning, *C. striata* were collected in four regions across this distribution and throughout their spawning season. By assessing energetic allocation (lipid, energy density and total energy) in muscle, liver and gonad tissues, *C. striata* were identified as mixed breeders because while they mobilized somatic energy stores towards reproductive development, they also used energy acquired from their diet to sustain reproductive output throughout the spawning season. Unlike male fish, female fish both invested more energy into liver and gonad tissues and exhibited regional differences in energetic values. For both sexes, *C. striata* in the northern portion of the distribution had lower energetic values both in the somatic stores and towards gonadal development than the fish in the southern portion of the distribution, possibly because of longer migration distance. Overall, the authors found significant spatial variation in energetic constraints that may affect reproductive output and success (recruitment), a relevant result as *C. striata* are a popular recreational and commercial species throughout this distribution.

## 1 INTRODUCTION

Fish reproductive strategies are diverse, ranging from single to multiple breeding opportunities, oocyte development timing (synchronous, group synchronous and asynchronous), spawning pattern (total and batch), fecundity type (indeterminate and determinate), fertilization (internal and external) and embryonic development (oviparity and viviparity) (Murua & Saborido‐Rey, 2003; Wootton, 1984). These reproductive strategies serve as alternative solutions to a pervasive problem of maximizing lifetime fitness through producing viable offspring while balancing energetically costly reproduction. In some cases, the energetic cost of reproduction can lead to semelparity (Kindsvater *et al*., 2016) or increase natural mortality in iteroparous species (Nielsen *et al*., 2012). In unpredictable environments, adults may forgo spawning or produce fewer batches of eggs if spawning conditions are poor (McBride *et al*., 2015) to increase survival probability into the next spawning season (Finstad *et al*., 2002). Therefore, energy allocation patterns for a particular species can provide insights into population dynamics and spawning‐related life‐history traits that are useful to understand from both an ecological perspective and fisheries management standpoint.

Energy is supplied through the diet and allocated towards maintenance, with any remaining surplus available for growth, storage and reproduction (for mature individuals). Maintenance supports routine metabolic processes which include standard metabolic rates and metabolism used for daily activities such as swimming and feeding (Treberg *et al*., 2016). Growth, and subsequently larger body sizes, can be advantageous for multiple reasons including the ability to store more energy for overwintering (Bunnell & Marschall, 2003), higher swimming efficiency (Nøttestad *et al*., 1999) and increased fecundity (Hixon *et al*., 2014). Surplus energy can be stored, typically in the form of lipids, within the liver or muscle tissue (Brown & Murphy, 2004), but sometimes in the viscera (Cook *et al*., 2021) and skin (Jørgensen *et al*., 1997). Stored lipids provide a direct source of energy that can be mobilized to other regions of the body when needed, as is done during reproductive development. Specifically, for female fish, liver energy storage is important because vitellogenin, a lipoprotein synthesized in the liver, is mobilized towards the gonad for egg development (Hiramatsu *et al*., 2002). Energy storage sites can be species‐specific (Fiorin *et al*., 2007), and sometimes distinctive energy allocation strategies can lead to a noticeable difference in reproductive output in terms of both egg quality and quantity (dos Santos *et al*., 2010). Thus, energy storage allows energy acquisition (feeding) and usage (spawning) to be separated in time and/or space, which is particularly important in environments with pulsed productivity.

The source of energy partitioned towards reproductive development varies across reproductive strategies. Fish that mobilize energy from stored reserves are classified as capital breeders, whereas fish that supply energy through food intake are identified as income breeders; some fish use both strategies and are classified as mixed breeders (McBride *et al*., 2015). Capital breeders supply a substantial percentage of their stored somatic energy towards spawning, leaving them energy deplete at the end of the spawning season (Dawson & Grimm, 1980; Jørgensen *et al*., 1997). On the other end of the spectrum, income breeders do not use energy stores from somatic reserves (Domínguez‐Petit *et al*., 2010), and their reproductive output can be affected by the food supply during the spawning season (Basilone *et al*., 2020). Mixed breeders primarily acquire energy through their diet during the spawning season and also supplement energy from somatic stores, which exhibit a slight decline throughout spawning (Aristizabal, 2007; Burns & Fuiman, 2020). These differing breeding strategies impose a range of energetic constraints towards reproduction.

Spawning grounds provide a suitable habitat for larval hatching and rearing, increasing the chance of offspring survival (Jørgensen *et al*., 2008). Nonetheless, some fish undergo prespawning migrations because conditions at spawning grounds may not be optimal for adults year‐round for overwintering and/or because of separation from feeding grounds (Alexander, 1998; Buehler & Piersma, 2008). While ultimately advantageous, prespawning migrations can be energetically costly and can reduce the energy available for egg production (Hendry & Berg, 1999). Some species show a spectrum of iteroparity to semelparity across varying migration distance where fish with longer migrations have a greater propensity towards semelparity because of the combined cost of migration and reproduction (Glebe & Leggett, 1981). In some cases, migrating fish prepare by storing more energy before migration (Gaillard *et al*., 2015). Under this premise, larger fish have an advantage because of their greater energy storage capacities and higher swimming efficiencies (Jørgensen *et al*., 2008; Slotte, 1999). Therefore, migration serves as an important life‐history trait that can be considered a secondary reproductive cost, and the impacts of migration distance can be explored through energy allocation dynamics.

For fish species that inhabit a wide distributional range, in addition to gradients in migration distance, differences in reproductive energy allocation and somatic energy storage can occur across depth (Hoey *et al*., 2007), temperature (Feiner *et al*., 2016) and/or latitudinal gradients (Mollet *et al*., 2013). Energy allocation can also be affected by overwintering preparation, where individuals at higher latitudes with pulsed environmental productivity rapidly acquire energy reserves, whereas fish at lower latitudes with constant productivity accumulate them more gradually (Schultz & Conover, 1997). Density dependence across a range can also influence energy allocation where fish in densely populated habitats exhibit lower reproductive investment and slower growth (Pritt *et al*., 2020). Investigating how energy allocation differs across the wide distribution of certain fish species can provide insight into potential differences in reproductive output and recruitment success throughout a distribution.

Black sea bass (*Centropristis striata*) are an economically and ecologically important fisheries species managed as three genetically distinctive stocks: the Gulf of Mexico stock, the Southeastern stock (SES) located from Eastern Florida to Cape Hatteras, NC, and the Northern stock (NS) located from Cape Hatteras, NC to the Gulf of Maine (Bowen & Avise, 1990; McCartney *et al*., 2013). Because of the physical dynamics of the Gulf Stream current (Gray & Cerame‐Viuas, 1963), the tip of Cape Hatteras, NC, acts as a biological barrier leading to genetic and demographic variation between the SES and NS of *C. striata* (Roy *et al*., 2012). The NS of *C. striata* (hereafter referred to as NS *C. striata*) inhabits a wide latitudinal inshore distribution, spanning *c*. 6° of latitude during the summer spawning season, and migrate offshore towards the southeastern continental shelf edge to overwinter (Musick & Mercer, 1977) in a narrower range of latitude. This migration pattern results in some fish migrating a farther distance than others, and because NS *C. striata* exhibit a high degree of site fidelity (Moser & Shepherd, 2008), these life‐history differences can persist throughout regional sub‐groups of NS *C. striata*. In addition, latitudinal effects (*i.e*., seasonality and/or temperature) have led to differences in the initiation, duration and reproductive output during the spawning season (Slesinger *et al*., 2021). NS *C. striata* are also protogynous hermaphrodites (Mercer, 1978; Wenner *et al*., 1986), further complicating an understanding of life‐history variation with respect to lifetime reproductive output. The potential misspecification in stock assessment models by not accounting for within‐stock life‐history variation of NS *C. striata* has prompted concerns. A recent stock assessment report advised that NS *C. striata* should be split into two management sub‐groups at the Hudson Canyon because of differing *C. striata* life‐history characteristics (NEFSC, 2017).

An investigation into the intraspecific differences of NS *C. striata* energy allocation throughout their distribution will provide information pertinent to fisheries management and regional ecosystem dynamics. First, fisheries management plans are based on estimated biomass for state‐specific quotas, and additional insight into *C. striata* spawning and recruitment can aid in future management plans. Second, the U.S. Northeast Shelf has been experiencing rapid ocean warming (Chen *et al*., 2020; Pershing *et al*., 2015); regional variation in energy allocation provides a base onto which the current and future effects of ocean warming can be anticipated. Third, *C. striata* centre of biomass has been shifting northward over time (Bell *et al*., 2015; Kleisner *et al*., 2017), which could be a response to ocean warming in the southern portion of their range (Slesinger *et al*., 2019) and/or increased biomass in the northern region as a result of previous fisheries management (Bell *et al*., 2015). Heterogeneity in energy allocation may reveal that life‐history strategies at the expanding edge differ from those closer to the centre of biomass. Therefore, the authors of this study asked: (a) are there regional differences in NS *C. striata* somatic and reproductive energetics, and (b) what are the seasonal trends in energetic usage throughout the spawning season across the entire distribution?

## 2 MATERIALS AND METHODS

### 2.1 Collection and sample processing

**FIGURE 1:**
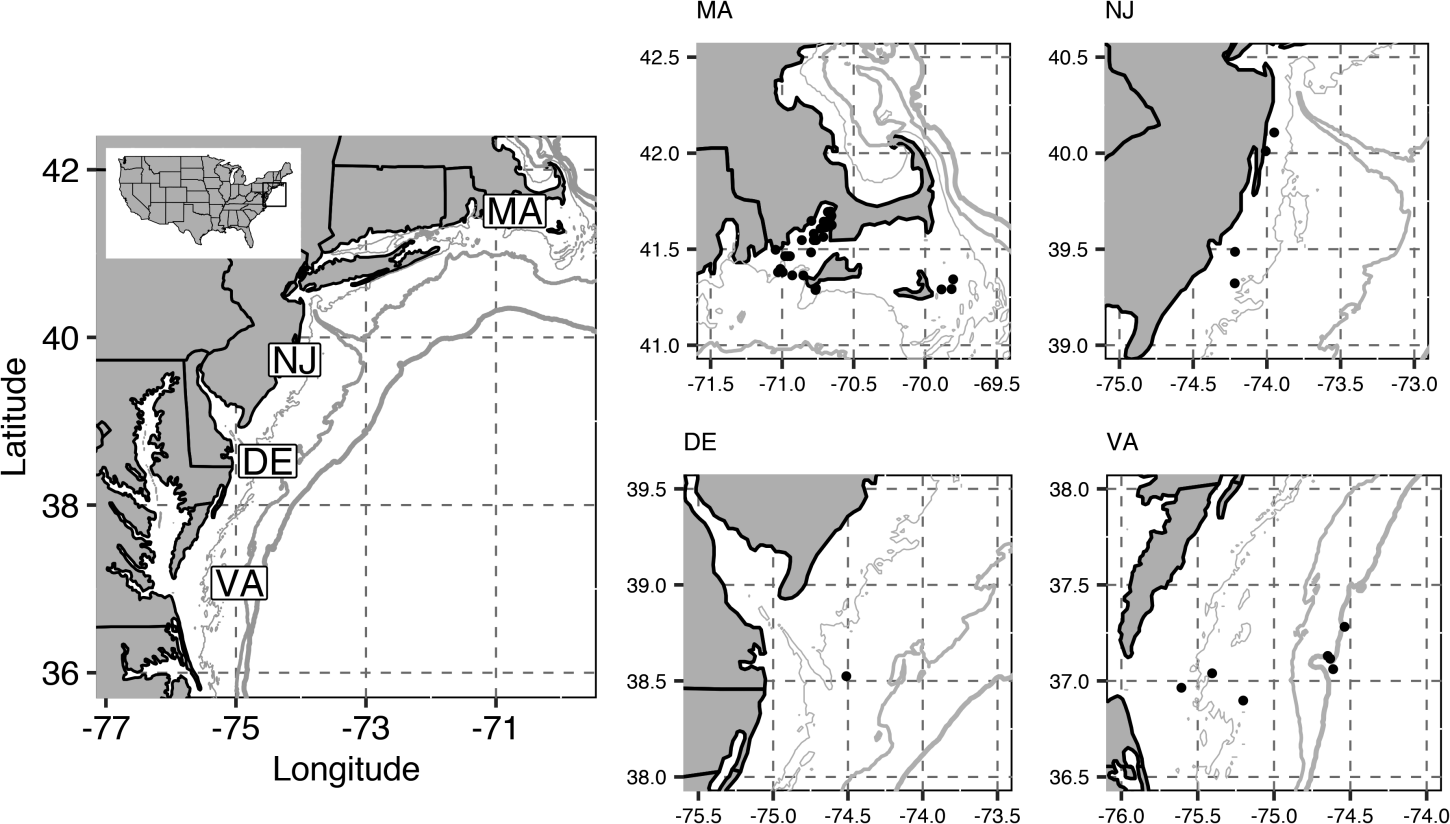
Collection locations of *Centropristis striata*. Map of sampling locations of *C. striata*, modified from Slesinger *et al*., 2021. Left panel shows the rough locations of sampling by each region code (VA, DE, NJ and MA), and the right panel shows each sampling location for each region. For each panel, the grey lines indicate the 25, 50 and 100 m isobaths from thin to thick lines. The 100 m isobath also represents the continental shelf break for the U.S. Northeast shelf

Collections and sample processing of NS *C. striata* are more fully described in Slesinger *et al*. (2021). Briefly, NS *C. striata* were collected across their distribution, from south to north, off the coasts of Virginia (VA), Delaware (DE), New Jersey (NJ) and Massachusetts (MA) using both hook and line and fish traps (Figure 1). Collections occurred in 2018 and 2019, and targeted the spawning season which occurs from *c*. April to October (Drohan *et al*., 2007). After collection, fish were measured to obtain a length and weight, and dissected to remove the liver and gonad. For both, a wet‐weight (±0.01 g) of the entire organ was measured before processing. A section of epaxial muscle tissue (*c*. 3.35 ± 1.163 g) was also removed above the lateral line and underneath the first dorsal spine. For each tissue, a weighed sub‐sample was preserved at −80°C for lipid extractions.

The sex and maturity stage of each fish were determined by a macroscopic inspection of the gonad to link energetic analyses with spawning condition. Maturity stages were classified as developing, spawning capable (includes ripe and ripe and running fishes), spent (regressing) and resting (regenerating) based on classifications from Brown‐Peterson *et al*. (2011). Because *C. striata* are protogynous hermaphrodites (Mercer, 1978), transitioning fish were identified using criteria from Klibansky and Scharf (2015) and removed from analysis (*n* = 5).

### 2.2 Ethics statement

A detailed description of fishing permits and IACUC protocol have been published in Slesinger *et al*. (2021). Slesinger performed all hook‐and‐line collections and euthanasia, which were done in accordance to Rutgers University IACUC Protocol (#PROTO201800054). Additional state‐ or federal‐issued fishing permits can be found in Table 1 of Slesinger *et al*. (2021).

### 2.3 Lipid extractions

A sub‐sample of the total number of *C. striata* collected (281 of 898) was selected for lipid extractions to provide *c*. 5–8 fish per day of collection per region, covering a range of maturity stages, sexes and sizes (Table 1). A similar size range for female and male fish was also attempted to limit confounding sex and size variation known in this species (Supporting Information Figure S1). For each fish, the total lipids were extracted from liver, gonad and muscle tissues for a total of 843 lipid extractions. Before lipid extractions, *c*. 0.5 g sample of tissue was weighed (±0.001 g) to provide a wet weight (WW), freeze dried to a constant weight, and then reweighed to provide a dried weight (DW) and calculate the % dry‐weight (%DW). To run samples in duplicate, the dried sample was then homogenized through mechanical crushing, divided into two equal parts and reweighed to provide a dried weight of each sub‐sample. Total lipids were extracted using a modified chloroform:methanol extraction (Folch *et al*., 1957). On each extraction day, an external standard (nonadecanoic acid, C19:0) was also run in duplicate. For each sample, the lipid extraction process was repeated thrice to ensure high extraction efficiency. Total lipids were measured gravimetrically as the weight gain in a pre‐weighed gas‐chromatography vial and expressed as lipid concentration (LC; g lipid g^−1^ DW) for each tissue type (muscle: MLC, liver: LLC, gonad: GLC). If the difference in LC between samples run in duplicate was >10%, a third sample was analysed. The mean of replicate samples for each fish was used for analyses below.

**TABLE 1:** Sample sizes of fish by region, sex and maturity stage

Region	Sex	Maturity stage				*n*
		DEV	SPC	SPT	REST	
MA	F	10	23	6	5	44
	M	5	10	7	1	23
NJ	F	23	18	6	4	51
	M	13	9	5	3	30
DE	F	9	17	14	0	40
	M	7	15	17	1	40
VA	F	4	16	7	0	27
	M	8	7	11	0	26

*Note. N* is the total number of fish per region by sex. For maturity stage, DEV: developing, SPC: spawning capable, SPT: spent and REST: resting.

### 2.4 Estimation of energy densities

To estimate the energy density (ED; kJ g^−1^ WW) of each tissue, both lipid and protein concentrations are required. LC and %DW were measured in the present study, whereas the ED of each tissue type (muscle: MED, liver: LED, gonad: GED) was estimated with and validated using proximate composition data from the ED values reported in Wuenschel *et al*. (2013) as follows. The %DW from each sample provided the total g DW per g WW. Lipid (LC), protein (PC) and ash (AC) concentration, all as g g^−1^ DW, were assumed to comprise all the DW (there is minimal energetic contribution from carbohydrates in fish; Love, 1980) and therefore add to one. Tissue‐specific linear relationships between AC and %DW from the Wuenschel *et al*. (2013) samples were used to estimate AC from the %DW of the samples in the present study. Protein concentration was calculated as one minus the combined LC and estimated AC. Next, LC and PC were separately multiplied by the respective tissue %DW to provide lipid (LC_w) and protein concentration per g WW (PC_w). Finally, LC_w and PC_w were converted to an ED by multiplying 39.565 kJ g^−1^ lipid and 23.64 kJ g^−1^ protein, respectively (Henken *et al*., 1986); total ED of the tissue was the sum of the lipid and protein EDs. To validate the ED estimates to Wuenschel *et al*. (2013), a linear regression of ED by  %DW was used to assess the fit of both data sets together (Supporting Information Figures S2–S4). The total weights of the liver and gonad were multiplied by their respective ED to obtain a liver total energy (LTE; kJ) and gonad total energy (GTE; kJ). A complete list of abbreviations and equations is presented in Table 2.

**TABLE 2:** Abbreviations and short equations used throughout the text

Abbreviation	Term	Equation	Units
%DW	Per cent dry weight	=100*(DW÷WW)	%
DW	Dried weight	–	g
ED	Energy density	=P_ED + L_ED	kJ g^−1^ WW
AC	Ash concentration	=g ash÷g DW	g g^−1^ DW
LC	Lipid concentration	=g lipid÷g DW	g g^−1^ DW
LC_w	Lipid concentration (wet)	=LC*(%DW÷100)	g g^−1^ WW
L_ED	Lipid energy density	=LC_w*39.565 kJ g^−1^ lipid	kJ g^−1^ WW
PC	Protein concentration	=1 – (LC + AC)	g g^−1^ DW
PC_w	Protein concentration (wet)	=PC*(%DW÷100)	g g^−1^ WW
P_ED	Protein energy density	=PC_w * 23.64 kJ g^−1^ protein	kJ g^−1^ WW
WW	Wet weight	–	g
GED	Gonad energy density	–	kJ g^−1^ WW
GLC	Gonad lipid concentration	–	g g^−1^ DW
GTE	Gonad total energy	–	kJ
LED	Liver energy density	–	kJ g^−1^ WW
LLC	Liver lipid concentration	–	g g^−1^ DW
LTE	Liver total energy	–	kJ
MED	Muscle energy density	–	kJ g^−1^ WW
MLC	Muscle lipid concentration	–	g g^−1^ DW

*Note*. Tissue‐specific abbreviations are at the end of this table.

### 2.5 Data analysis

All data analysis was performed in R (Version 4.0.1; R Core Team, 2019). Generalized linear models (GLMs) with beta distribution were run using the package Betareg (Cribari‐Neto & Zeileis, 2010), and GAMs were run using the package MGCV (Wood, 2013). Significance of a value was determined at *α* = 0.05.

#### 2.5.1 Tissue‐specific lipid and energy

Compositional measurements [LC, ED and total energy (TE)] were analysed for muscle, liver and gonad tissue using GLMs. Sex‐specific (female, male) models were run separately instead of including sex as an interactive factor given different energy allocation in these tissues by sex and because an interaction term would provide information on how the main effect of sex may or may not differ with each predictor, rather than provide sex‐specific information on the effect of a predictor on the response variable. Nonetheless, before analysis, each compositional measurement for each tissue was first assessed as to whether sex was an important factor in the main models. If sex was an important factor, the GLMs were run separately, and if not, sex was combined. Preliminarily analyses showed similar sex‐specific energetics in muscle, but not for liver and gonad.

The GLMs were used as an explanatory tool to evaluate the importance of region for each compositional measurement rather than to identify the “best” fit models to explain variation in the response variables. As such, a null model with predictor variables of *weight* (continuous) and *maturity stage* (categorical with four levels) was compared with a regional model with the same predictor variables as in the null model and the addition of *region* (categorical with four levels). BIC, which provides robust hypothesis testing without overfitting, was used to evaluate whether region should be included in each model (Aho *et al*., 2014). An error distribution and appropriate link function were chosen for each compositional measurement to account for specific data structure (Supporting Information Table S1). A beta distribution with a logit link was used for the LC models because these values are between 0 and 1. A gamma distribution was used for both the ED and TE models because both metrics are non‐zero with a right tailed distribution. The canonical inverse link was used for the ED models, and a log link was used for the TE models because of a better fit.

#### 2.5.2 Seasonal patterns

Generalized additive models (GAMs) were used to further assess *C. striata* TE usage throughout the spawning season. A Julian day integer was obtained based on collection date of the fish. Separate sex‐specific GAMs were run for response variables LTE and GTE with *weight* (continuous) and *region* (factor with four levels) as parametric predictors and a smooth term of *day* with a *region* interaction as the non‐linear term. For the smooth term, a thin plate spline was used. K, the number of knots, was selected through BIC and checked using gam. check() in the MGCV package in R to ensure enough knots for analysis while avoiding overfitting.

Results from these GAMs provided a continuous prediction of sex‐specific LTE and GTE per region throughout the spawning season. To compare LTE and GTE across region, a median weight of all fish (315 g) was used for predictions. The day of the start and end of spawning, which were taken from the criterion of 25% of the population being spawning capable, and peak gonado‐somatic index were obtained from Slesinger *et al*. (2021). From these, the LTE and GTE were estimated for those days to provide total energy present at the start and throughout to the end of spawning. Cumulative energy present throughout spawning over the spawning duration and the energy usage per day were calculated based on the cumulative energy and spawning duration.

## 3 RESULTS

### 3.1 Tissue‐specific lipid and energy

In total, 14 GLMs were analysed which included 2 for muscle (LC and ED), 6 for liver and 6 for gonad (sex‐specific LC, ED and TE). Of the 14 GLMs, region was an important predictor for 10 of the models (Table 3). For muscle, region was important for MED but not for MLC. For female fish, region was always an important predictor, whereas for male fish region was an important predictor for LLC, LTE and GLC. For all model selections, ΔBIC was >2 (Supporting Information Table S2).

**TABLE 3:** Model results and coefficients for the regional GLMs

Tissue	Sex	Model	n	Intercept	Weight	SPC	SPT	REST	DE	NJ	MA
Muscle	All	LC	281	**−2.739**	**0.000**	**−0.078**	**−0.157**	**−0.220**	–	‐	–
		ED	281	**0.194**	0.000	**0.005**	**0.007**	−0.002	−0.003	**0.012**	**0.014**
Liver	F	LC	162	**−0.524**	0.000	**−0.243**	**−0.574**	0.012	−0.046	**−0.326**	**−0.660**
		ED	162	**0.114**	0.000	**0.016**	**0.017**	−0.009	0.001	**0.019**	**0.026**
		TE	162	**3.065**	**0.003**	**−0.212**	**−0.670**	**−0.404**	−0.058	**−0.425**	**−0.522**
	M	LC	119	**−0.749**	0.000	0.055	0.017	0.140	**−0.386**	**−0.452**	**−0.552**
		ED	119	**0.122**	0.000	0.003	0.003	0.009	–	‐	‐
		TE	119	**2.945**	**0.002**	−0.214	**−0.299**	**−0.732**	−0.229	**−0.459**	**−0.540**
Gonad	F	LC	162	**−1.414**	0.000	0.055	**−0.505**	**−0.912**	**0.153**	**−0.176**	0.006
		ED	162	**0.137**	0.000	**0.020**	**0.043**	**0.062**	−0.004	**0.021**	**0.015**
		TE	162	**3.744**	**0.003**	0.134	**−1.742**	**−2.589**	0.161	**−0.456**	**−0.603**
	M	LC	119	**−1.826**	0.000	0.000	0.045	−0.124	**0.171**	**0.174**	**0.182**
		ED	119	**0.275**	0.000	−0.003	**−0.033**	**−0.069**	‐	‐	‐
		TE	119	**3.157**	**0.001**	0.171	**−0.992**	**−2.830**	‐	‐	‐

*Note*. Statistically significant coefficients are bolded. “‐” indicates region was not selected for in the specific model. The intercept is set as developing for maturity stage and VA for region. For maturity stage, SPC: spawning capable; SPT: spent and REST: resting. GLM: generalized linear model.

#### 3.1.1 Muscle

**FIGURE 2:**
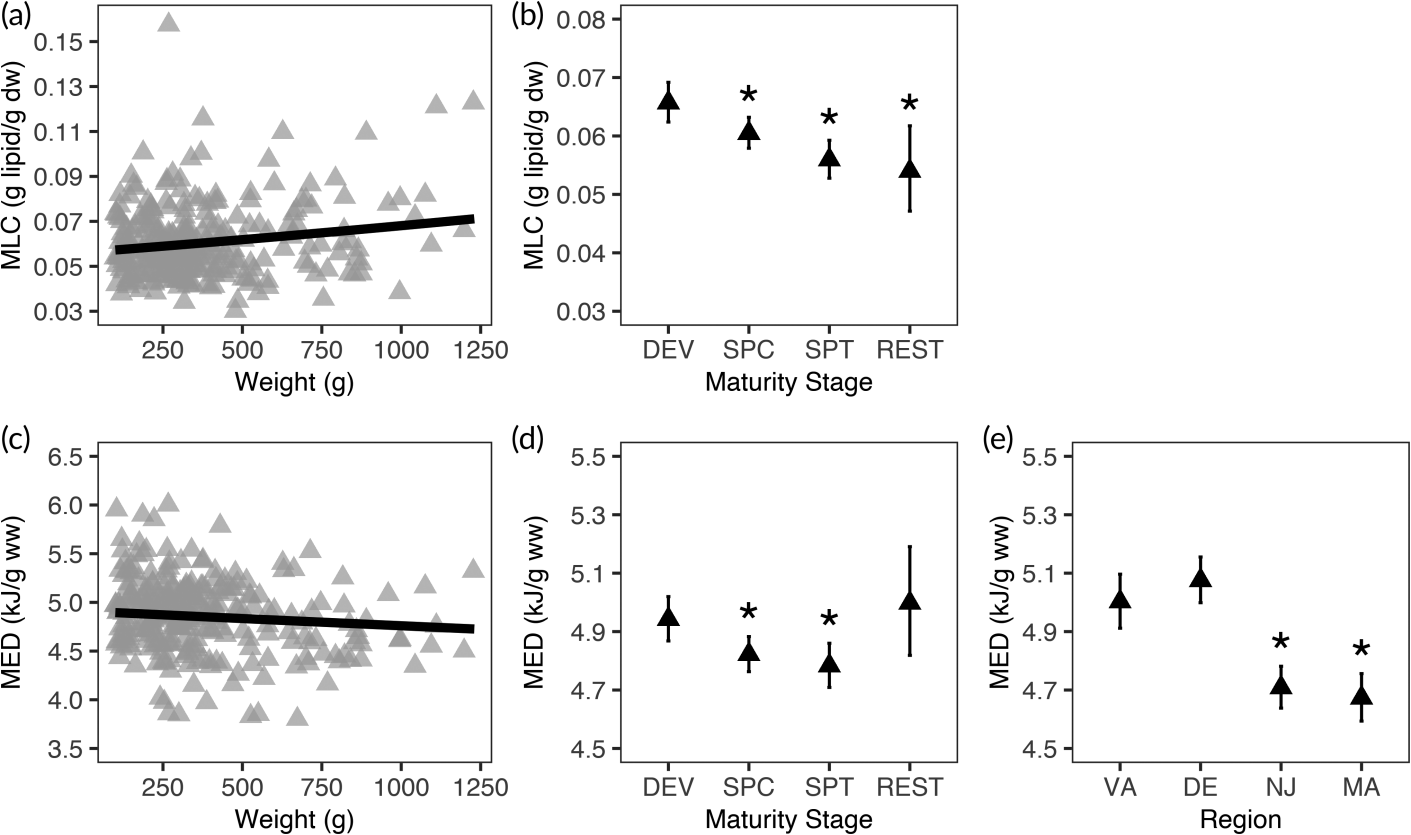
Compositional generalized linear model (GLM) results for muscle. Compositional model results from the muscle GLMs where results for MLC (a and b) and MED (c–e) are shown. The model fit of the partial residual weight by MLC (a) and MED (c) are shown by a black line with raw data points depicted in grey triangles. For MLC, the regression for weight is significant but is non‐significant for MED. The partial residuals of maturity stage (b and d) and region (e) are plotted in black triangles (mean ± s.e.). For maturity stage, DEV: developing, SPC: spawning capable, SPT: spent and REST: resting. For region, the region codes are plotted, from left to right, following the continental shelf from south to north. * indicates a significant difference (*P* < 0.05) from the model intercepts (DEV for maturity stage and VA for region). No regional results for MLC indicate that region was not selected for in the MLC model. Female and male fish are combined in these plots. Note the different *y*‐axis scales

Muscle GLMs combined female and male fish for analyses (Table 3). For MLC, region was not an important predictor. Weight was a significant predictor (*P* < 0.001), where MLC increased with weight (Figure 2a), and for maturity stage, spawning capable, spent and resting categories were all significant when compared to developing fish (*P* < 0.05; Figure 2b). For MED, the effect of weight was not significant (*P* > 0.05; Figure 2c), indicating variation in MED was not driven by the size of the fish. Spawning capable and spent fish were significant for maturity stage (*P* < 0.05; Figure 2d). Region was an important predictor for MED, where NJ and MA were significantly different from VA fish (*P* < 0.001; Figure 2e). Altogether MLC and MED declined as maturity stage advanced, and MED exhibited some postspawning recovery. Regionally, MED was lower in the northern sampling sites including NJ and MA.

#### 3.1.2 Liver

**FIGURE 3:**
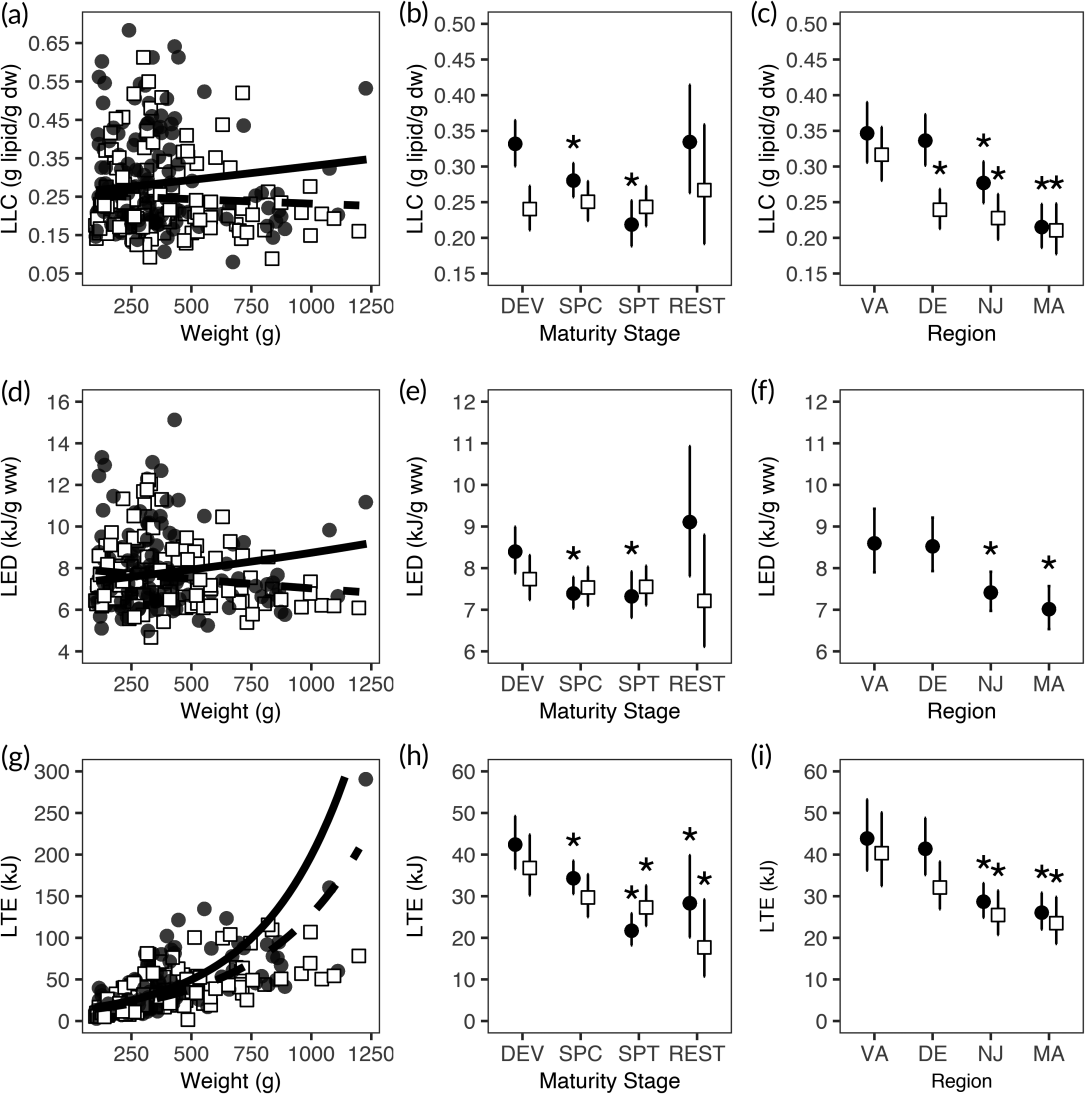
Compositional generalized linear model (GLM) results for liver. Compositional model results from the liver GLMs where results for LLC (a–c), LED (d–f) and LTE (g–i) are shown. The model fit of the partial residual weight for LLC (a), LED (d) and LTE (g) are plotted in a solid line for female fish and dashed line for male fish. The regression for weight is only significant for LTE for both sexes. The partial residuals of maturity stage (b, e, h) and region (c, f, i) are plotted in solid circles for female fish and open squares for male fish (mean ± s.e.). For maturity stage, DEV: developing, SPC: spawning capable, SPT: spent and REST: resting. For region, the region codes are plotted, from left to right, following the continental shelf from south to north. * indicates a significant difference (*P* < 0.05) from the model intercepts (DEV for maturity stage and VA for region). Male GLMs where region was not selected are shown by an absence of regional data points plotted for male fish. Note the different *y*‐axis scales for weight within each compositional measurement. Sex: (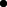) F, (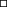) M

For female fish, liver energetics were clearly affected by spawning, and region was important for all measurements (Table 3). For LLC, the effect of weight was not significant (*P* > 0.05; Figure 3a), and spawning capable and spent fish were significantly different from developing fish (*P* < 0.05; Figure 3b). Regionally, NJ and MA were significantly different from VA (*P* < 0.01), but showed a general declining trend in LLC from south to north (Figure 3c). Similar to LLC, for LED there was a non‐significant effect of weight (*P* > 0.05; Figure 3d), and spawning capable and spent fish were significantly lower than in developing fish (*P* < 0.01; Figure 3e). Across region, LED was significantly lower (*P* < 0.01) in NJ and MA fish compared to VA fish (Figure 3f). For LTE, weight was a significant predictor (*P* < 0.001), with increasing weight leading to higher LTE (Figure 3g). As maturity stage advanced, there was a significant decline in LTE for spawning capable, spent and resting fish from developing fish (*P* < 0.05; Figure 3h). Across region, NJ and MA were significantly different from VA (*P* < 0.01), decreasing from south to north (Figure 3i). Altogether the size of the fish was only important for LTE, each compositional measurement decreased with advancing maturity stage, with some postspawning recovery seen by a slight increase in values from spent to resting fish, and values were generally higher in the southern locations of VA and DE.

Male fish liver energetics differed from female fish in that specific measurements were less affected by maturity stage, and region was not an important predictor for LED (Table 3). For LLC, the effect of weight and maturity stage were not significant (*P* > 0.05; Figures 3a,b), whereas the regional effect was significant with VA higher (*P* < 0.001) than the other regions (Figure 3c). Similar to LLC, there were no significant differences in LED with weight and between maturity stages (*P* > 0.05; Figures 3d,e). Similar to female fish LTE, weight was a significant predictor (*P* < 0.001; Figure 3g) and across region, LTE was significantly different in NJ and MA fish than from VA fish (*P* < 0.01; Figure 3i). LTE was the only measurement that was significantly affected by maturity stage where LTE decreased as maturity stage advanced, with spent and resting fish significantly lower than developing fish (*P* < 0.05; Figure 3h). This suggests a liver size contribution to total energy levels rather than a compositional change. As in female fish, the weight of the fish was only significant for LTE, indicating an allometric relationship between body size and liver size contributing to higher LTE. Region was important for male LLC mostly because of the higher values in the VA fish, but there was a general decline in LTE from south to north suggesting a liver size component as well (region was not an important predictor for LED).

#### 3.1.3 Gonad

**FIGURE 4:**
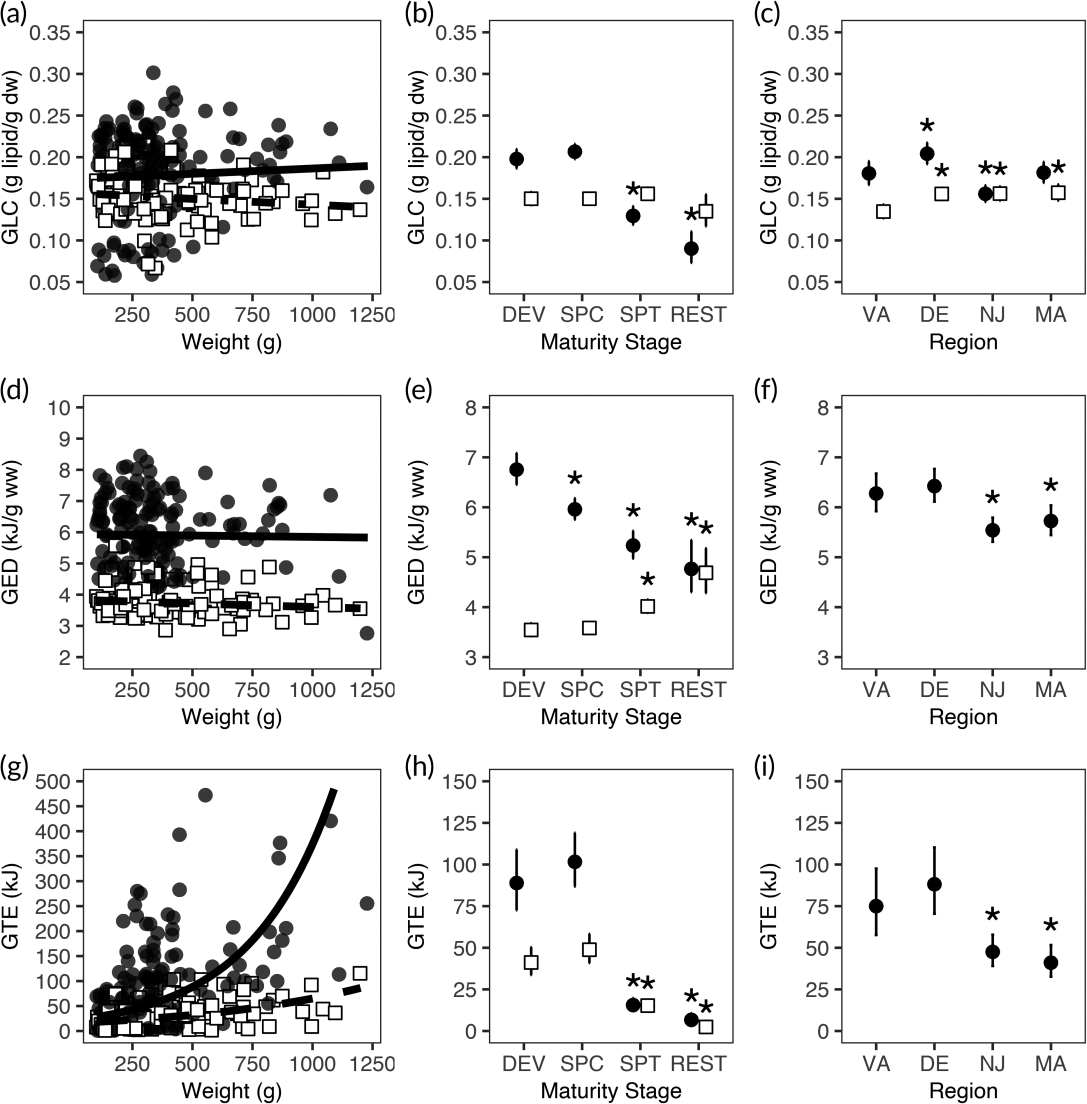
Compositional generalized linear model (GLM) results for gonad. Compositional model results from the gonad GLMs where results for GLC (a–c), GED (d–f) and GTE (g–i) are shown. The model fit of the partial residual weight for GLC (a), GED (d) and GTE (g) are plotted in a solid line for female fish and dashed line for male fish. The regression for weight is only significant for GTE for both sexes. The partial residuals of maturity stage (b, e, h) and region (c, f, i) are plotted in solid circles for female fish and open squares for male fish (mean ± s.e.). For maturity stage, DEV: developing, SPC: spawning capable, SPT: spent and REST: resting. For region, the region codes are plotted, from left to right, following the continental shelf from south to north. * indicates a significant difference (*P* < 0.05) from the model intercepts (DEV for maturity stage and VA for region). Male GLMs where region was not selected are shown by an absence of regional data points plotted for male fish. Note the different *y*‐axis scales for weight within each compositional measurement. Sex: (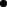) F, (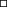) M

Region was important for all female gonad measurements (Table 3). GLC was not significantly affected by weight (*P* > 0.05; Figure 4a), but for maturity stage, GLC significantly declined in the spent and resting stages (*P* < 0.001; Figure 4b). Across region, DE and NJ were significantly different from VA (*P* < 0.05; Figure 4c). For GED, the effect of weight was not significant (*P* > 0.05; Figure 4d). Spawning capable, spent and resting fish were significantly different from developing fish (*P* < 0.001; Figure 4e), and throughout region, NJ and MA were significantly different from VA (*P* < 0.05; Figure 4f). For GTE, weight was a significant predictor (*P* < 0.001) with larger fish predicted to have higher GTE (Figure 4g). With advancing maturity stage, there was a significant decline in spent and resting fish (*P* < 0.001; Figure 4h), and by region there was a significant difference in NJ and MA from VA fish (*P* < 0.01; Figure 4i). Altogether as maturity stage advanced, GLC, GED and GTE declined. Nonetheless, between developing and spawning capable stages, there was no change in GLC, a decrease in GED and an increase in GTE. This suggests that between these two stages, GLC of dried tissue remained constant while a decline in GED could be driven by the hydration of oocytes (increasing the denominator), and an increase in GTE a result of increasing gonad size. For the regional trends, there was also a general decline in gonad energetics from south to north.

Male fish gonad energetics were generally lower than female fish and region was only important for GLC (Table 3), where VA was significantly lower than the other regions (*P* < 0.001; Figure 4c). Similar to female fish, weight was only a significant predictor for GTE (*P* < 0.001; Figure 4g). With advancing maturity stage, there was no change in GLC (Figure 4b), spent and resting fish GED was significantly different from developing fish (*P* < 0.001; Figure 4e), and there was a significant difference in spent and resting fish GTE from developing fish (*P* < 0.001; Figure 4h). Overall, male gonad energetics were largely unaffected by the predictors tested in the GLMs of this study, except for the significant effects of weight and maturity stage on GTE.

### 3.2 Seasonal patterns

The four seasonal GAMs (sex‐specific LTE and GTE) showed regional differences in energy usage throughout the spawning season. All plotted GAM results were predicted at the median weight (315 g) of all fish in the study to allow comparisons between regions and sex with the effect of weight removed. For all GAMs, weight was a significant predictor (*P* < 0.001) and explained a substantial, but not all, of the deviance in the models (Table 4). Nonetheless, the effect of weight was greater in LTE than GTE for both female and male fish. For LTE, the female and male GAMs explained 70.6% and 59.9% of the deviance, respectively (Table 4). The daily smooth terms for VA, DE, NJ and MA were significant (*P* < 0.01) for female fish and the VA daily smooth term was significant (*P* < 0.05) for male fish. Except for MA, all regional EDF values were near one suggesting a linear relationship between LTE and Julian day; MA exhibited a postspawning recovery and increase in LTE towards the end of the spawning season. For GTE, the female and male GAMs explained 79.7% and 72.3% of the deviance, respectively. For both, the smooth term for Julian day by region was significant for all regions (*P* < 0.001), and only GTE for male fish from VA showed a linear relationship.

**TABLE 4:** GAM results for each model with the coefficients for parametric values and estimated degrees of freedom (EDF) for smooth terms, where significant values (*P* < 0.05) are in bold

iModel	Term	VA	DE	NJ	MA	Weight	Dev. exp	Dev. exp without weight
LTE female	Coef	**2.623**	0.214	−0.199	−0.069	**0.003**	70.6%	28.3%
	EDF	**1.160**	**1.000**	**1.392**	**1.925**	‐		
LTE male	Coef	**2.564**	−0.012	**−0.298**	−0.098	**0.002**	59.9%	12.3%
	EDF	**1.276**	1.078	1.440	1.775	‐		
GTE female	Coef	**2.710**	**0.951**	0.143	**−0.753**	**0.003**	79.7%	57.2%
	EDF	**2.758**	**2.455**	**2.749**	**2.008**	‐		
GTE male	Coef	**2.698**	**0.283**	−0.189	**−0.702**	**0.002**	72.3%	56.1%
	EDF	**1.000**	**1.956**	**1.972**	**1.661**	‐		

*Note*. Both the deviance explained from the full model and full model without weight are reported to show relative influence weight has on total deviance explained. For the parametric terms, VA is the intercept.

**FIGURE 5:**
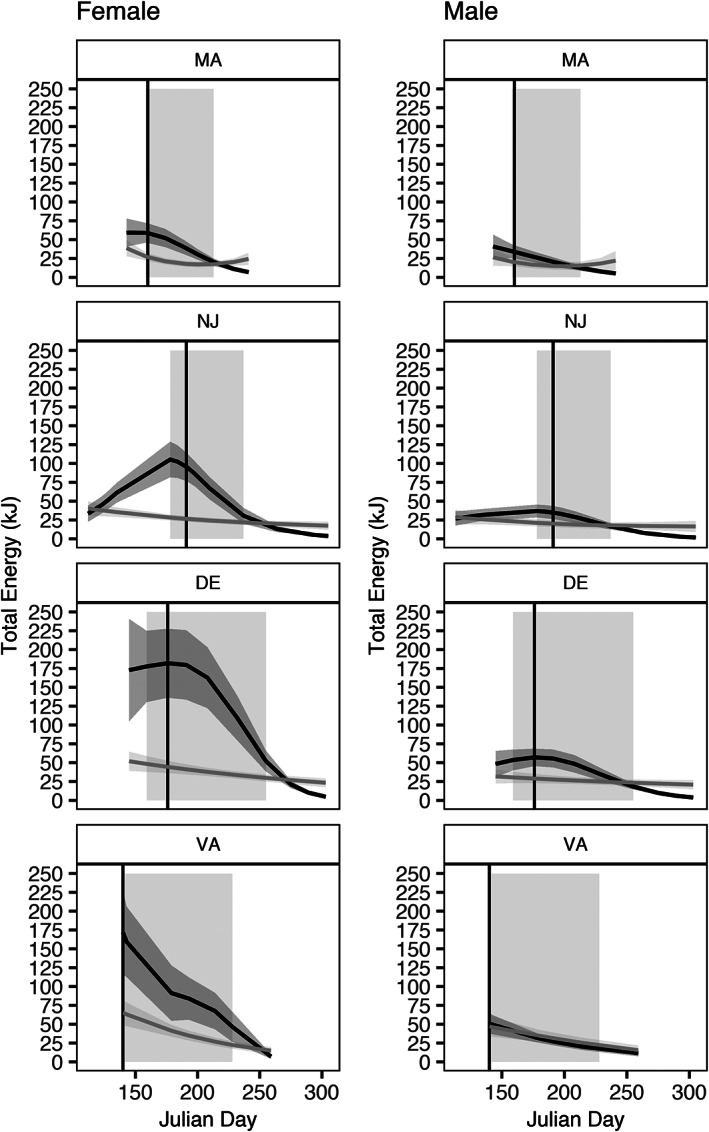
Seasonal GAM results for LTE and GTE. Seasonal GAM results plotted by region where LTE (grey line) and GTE (black line) are shown, the shading is the 95%c.i. and the left column are female fish and right column are male fish. The shaded grey rectangle denotes the length of the spawning season and the solid black vertical line shows the day of peak GSI from Slesinger *et al*. (2021). For reference, a Julian day of 150 is 30 May and 250 is 7 September. Tissue: (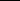) GTE, (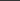) LTE

Across the spawning season, GTE was higher than LTE for female fish, whereas GTE and LTE were similar for male fish (Figure 5). LTE steadily declined throughout spawning, whereas GTE peaked or plateaued before declining. By incorporating the start and end of spawning days, and peak GSI day for each region from Slesinger *et al*. (2021), energy usage throughout the spawning season was assessed with respect to differing spawning season lengths between the regions. For each region, the decline in GTE occurred near or on the peak GSI day, and GTE and LTE intersected near the end of spawning (Figure 5). Regionally, there were higher starting GTE values in DE and VA, and lower in NJ and MA. The LTE cumulative kJ for female fish was higher in VA and DE (*c*. 3500 kJ) compared to NJ and MA (*c*. 1000 kJ). Female and male LTE cumulative kJ increased from north to south (Table 5). For GTE, cumulative kJ was highest in DE (female: *c*. 14,000 kJ; male: *c*. 4200 kJ) and lowest in MA (female: *c*. 2300 kJ; male: *c*. 1300 kJ). Although the spawning duration was longer in DE compared to MA, contributing to the higher cumulative kJ values, the kJ day^−1^ showed that the daily output of MA fish was *c*. 70% lower than DE.

**TABLE 5:** Liver and gonad total energy (kJ) at the start and end of spawning based on 25% spawning capable criterion in Slesinger *et al*. (2021)

Tissue	Sex	Region	Start (kJ)	End (kJ)	Cumulative kJ	Spawning days	kJ day^−1^
Liver	F	MA	27.41	17.09	1107.32	55	20.13
		NJ	28.10	21.77	1479.18	39	37.93
		DE	48.11	29.65	3700.19	96	38.54
		VA	64.48	22.11	3566.46	88	40.53
	M	MA	20.44	14.92	913.79	55	16.61
		NJ	21.12	17.75	1150.52	39	29.50
		DE	30.37	23.33	2576.53	96	26.84
		VA	46.85	21.70	2982.18	88	33.89
Gonad	F	MA	58.85	19.52	2312.87	55	42.05
		NJ	104.97	29.67	4137.46	39	106.09
		DE	182.16	49.94	13,848.58	96	144.26
		VA	167.36	44.80	8296.47	88	94.28
	M	MA	34.19	12.02	1270.37	55	23.10
		NJ	36.79	16.22	1686.80	39	43.25
		DE	57.12	17.77	4245.60	96	44.23
		VA	50.39	17.00	2738.17	88	31.12

*Note*. Values are estimated from the seasonal GAMs. Cumulative kJ is the total energy from start to end of spawning; spawning days: the length of the spawning season based on above criterion; kJ day^−1^ provides a crude estimate of the energy usage per day for the given tissue, sex and region.

## 4 DISCUSSION

There were significant sex‐specific changes in *C. striata* energetics throughout the spawning season as well as spatial variation across the NS for a majority of the measures evaluated. Throughout the spawning season, muscle, liver and gonad tissue energetic values declined, with some postspawning recovery in the liver and muscle, implying direct use of these tissues energy stores for spawning. Male fish invested less energy in reproduction than female fish and were also compositionally different as seen by size differences of the organ primarily influencing total energetic values. Female fish were more often dissimilar across their distribution than male fish, and there was a general pattern of lower energetics in the northern sampling locations (MA, NJ) compared to the southern sampling locations (DE, VA). Notably, less energy was allocated to gonad energy in the northern sampling sites, which is consistent with the lower GSI found in Slesinger *et al*. (2021). These energetic differences are significant. For example, in order for MA and DE female fish to have the same energetic output throughout the spawning season, MA fish would need to double the DE daily output to reach that value with a shorter spawning season.

NS *C. striata* energetic allocation in body stores and towards reproduction differed significantly across their distribution. In the muscle, MLC and MED decreased throughout spawning, suggesting some use of muscle energetics towards reproduction. Regionally, MED also decreased from south to north, which could be indicative of migration effects on prespawning conditions (Wuenschel *et al*., 2013), as NS *C. striata* migration distances are longer in the northern portion of the distribution. For the liver, female fish measurements all decreased throughout spawning with some postspawning recovery, whereas male fish measurements did not change, except for LTE. Regionally, the general decline in energetics from south to north was a dominant pattern for female fish but less so in male fish. For female fish, LLC, LED and LTE were all higher in the southern than the northern sampling regions, which would suggest a link between higher lipid storage leading to higher LED and LTE. For the male fish, only LLC and LTE were significant for region. Nonetheless, the regional effect for LLC was likely driven by the overall higher values in the VA fish compared to the rest of the fish, whereas LTE regional differences were similar to those seen in the female fish. Both female and male LTE were driven by liver size, but the authors suggest female livers were also compositionally different as seen by changes in LLC and LED, whereas male fish livers were compositionally similar as their total energetic differences were only driven by variances in liver size. Gonad energetics for female fish were similar to liver in that there was a comparable decreasing energetics trend from south to north and declines in gonad energetics throughout the spawning season. For the male fish, GLC was the only measurement where region was significant, and as in liver, this was likely driven by the differing values in VA fish compared to the rest. The increase in male GED was likely compositional where postspawning gonads contain less water decreasing the denominator, and a decrease in GTE was influenced by gonad size.

For both liver and gonad energetics, variance in these measurements increased with body weight for TE but not for LC and ED measurements (Figures 3a,d,g and 4a,d,g). The decreased variance in GLC and GED is likely related to an “inertial” effect where larger fish have greater general reserves and do not deplete these to the degree smaller fish may. The more dramatic increase in variance of GTE is a function of gonad size. On a seasonal basis, the maximum gonad size (during peak spawning) increases proportionally to fish size, and then after spawning, the gonad reduces to a very small size (*i.e*., a minimum weight near 0 g) for all fish, leading to minimal effect of fish size on resting gonad size. The liver also undergoes seasonal changes in size, albeit less dramatically, and the liver never reduces to such a small size in larger fish. As such, the range in liver size is more constrained in larger fish than it is for gonad size. The changes in variance in LLC and LED are less clear, and the increased variance of LTE is a function of liver variable composition and weight.

Seasonally, NS *C. striata* TE in liver and gonad decreased throughout spawning and maintained the trend of higher energetics in the southern sampling locations. Female GTE was higher than LTE throughout the spawning season and intersected at the end of spawning, whereas male fish GTE and LTE were similar throughout the spawning season. Cumulative LTE and GTE were both higher in female fish than male fish, but the net difference was higher in cumulative GTE (*e.g*., net difference for DE fish of 315 g between female and male was *c*. 2000 kJ for LTE and *c*. 10,000 kJ for GTE). The weight of the fish explained some of the deviance in the model, where increasing body size generally led to higher LTE and GTE, implicating an allometric effect of increasing body size leading to larger organs, and thus higher TE. Nonetheless, there was a greater effect of weight on LTE than on GTE. As mentioned above, although both organs undergo seasonal variation in size (and composition), the range of values in gonad weight is greater compared to liver weight, likely leading the weaker relationship. Throughout the spawning season, the day of peak GSI coincided with the decline in GTE. Regionally, cumulative LTE and GTE were lower in the northern than the southern sampling sites for both male and female fish. This difference could be a product of the longer spawning seasons in the southern sampling locations. Nonetheless, for MA fish with a shorter spawning season to match the cumulative GTE of DE fish, they would need to double their daily GTE kJ output of the DE fish (*c*. 280 kJ day^−1^). It should be noted that the cumulative energy metric assumes constant spawning frequency throughout the distribution, and increasing cumulative GTE could also be achieved in MA by increasing their spawning frequency. Whether this is possible given temperature regulation of oocyte development and/or liver energy storage or energy intake restraints is unknown, but warrants future investigation.

Throughout their distribution, NS *C. striata* energy allocation patterns suggest they use a mixed breeding strategy. Before spawning, NS *C. Striata* female fish LTE values ranged from 50 to 150 kJ, and LLC, LED and LTE values declined throughout the spawning season (Figures 3b,e,h). For capital breeding fish such as pouting (*Trisopterus luscus*), LTE values decreased throughout spawning, like in NS *C. striata*, but prespawning LTE values were closer to 400 kJ (Alonso‐Fernández & Saborido‐Rey, 2012). In contrast, LED values for European hake (*Merluccius merluccius*), an income breeder, remained constant throughout the spawning season (Domínguez‐Petit *et al*., 2010). For NS *C. striata*, Rosa *et al*. (2020) also found that hepatic lipid storage was mobilized towards gonad development, confirming liver energy consumption throughout the spawning season. Nonetheless, starting liver energetic values for NS *C. striata* were lower than in capital breeders, suggesting NS *C. striata* are using a mixed breeding strategy (Aristizabal, 2007). A mixed breeding strategy is conducive to the life history of NS *C. striata*, which are asynchronous multiple‐batch spawners with a long spawning season spanning from April to October (Drohan *et al*., 2007). Therefore, supplementing energy towards spawning through body stores relaxes the requirement of adequate energy intake through the diet, but also does not restrict the amount of available energy towards spawning to be determined months before spawning as *C. striata* migrate inshore.

Under the premise that NS *C. striata* are mixed breeders, the regional differences in energetic values of NS *C. striata* could be explained by different feeding conditions throughout the distribution. NS *C. striata* are generalist feeders that ingest a variety of prey consisting of fish, crustaceans, molluscs, bivalves and zooplankton (Garrison & Link, 2000; Steimle & Figley, 1996). Food limitation may not exist during the spawning season but energetic supply could be limited by nutritional quality. For example, NS *C. striata* in the Gulf of Maine had a less varied diet and, subsequently, lower condition than fish collected in Southern New England (McMahan *et al*., 2020). Variation in the diet or reliance on single prey items can lead to notable effects on fish nutrition. For example, salmonids experiencing thiamine deficiencies had higher instances of reproductive failure, and the thiamine deficiency was likely caused by consuming a diet primarily of clupeids (Fisher *et al*., 1996; Keinänen *et al*., 2018). In a spawning experiment, Southern flounder (*Paralichthys lethostigma*) fed a diet with high docosahexaenoic acid (an omega‐3 fatty acid) still supplemented their egg production with somatic stores, potentially because of a lack of other essential fatty acids in the diet (Burns & Fuiman, 2020). This is notable because *C. striata* dietary lipid and fatty acid intake has been shown to affect fertilization success and egg quality (Bentley *et al*., 2009). Altogether with some reliance on food supply during spawning, regional differences in diet could lead to changes in energy allocation and, subsequently, reproductive output.

A notable difference between spawning locations for NS *C. striata* is the relative distance required to migrate there from overwintering grounds located along the continental shelf edge of the southeastern portion of the U.S. Northeast shelf (Moser & Shepherd, 2008), leading to a gradient of minimal to no migration in the south to a longer migration (*c*. 400–500 km) to the north. In this study, NS *C. striata* in the northern locations began spawning at lower energy reserves, apparent in both muscle and liver tissues, which are suggested to be dominant energy stores for NS *C. striata* (Rosa *et al*., 2020; Wuenschel *et al*., 2013). In preparation for prespawning migrations, some fish store more energy to match the energetic demands of migration, which allows fish with differing migration distances to be energetically similar upon arrival to the spawning grounds (Gaillard *et al*., 2015; Hendry & Berg, 1999). In other cases, fish can allocate more energy towards reproduction upon arrival to the spawning grounds (Glebe & Leggett, 1981). Nonetheless, in the 2‐year period the authors studied, NS *C. striata* that migrate farther arrived at the spawning grounds with lower energy reserves and were unable to recover energy stores through the diet; these fish also had lower reproductive energy. Continued monitoring for NS *C. striata* in the northern portion of their distribution would provide additional insight into the frequency that fish enter spawning grounds in low condition and how this may impact reproductive development.

Differences in energy allocation across the distribution and between sexes may also be influenced by the fact that *C. striata* are protogynous hermaphrodites, changing sex from female to male. Male and female NS *C. striata* liver and gonad energetics differed substantially, whereas their muscle energetics were similar (and analysed together). Notably, similar muscle energy dynamics were not a size artefact, as similar size ranges were analysed between male and female fish. This suggests that the tissue energetics most impacted by spawning show a greater difference between the sexes, which is important to consider for a protogynous hermaphrodite. In the SES, *C. striata* reach peak fecundity at intermediate sizes instead of the largest sizes, likely because of a change in energetic allocation towards growth before sex transition (Klibansky & Scharf, 2017), which has also been found for other protogynous fish species (Gamboa‐Salazar *et al*., 2020). Peak fecundity at intermediate sizes contrasts with the typical relationship of increasing female size associated with higher fecundity (Hixon *et al*., 2014). Therefore, relationships between growth and reproduction may differ as fish age because these dynamics suggest that at some point fish shift a higher proportion of energy allocation towards growth and/or storage instead of reproduction, an advantageous tactic after transitioning to male. Nonetheless, NS *C. striata* typically transition sex after the spawning season when the ovary has reduced in total size and energy rather than leading up to or during the spawning season (Provost et al., 2017), so changes in energetic allocation are representative for the observed sex and not influenced by a loss of females in the system. The impacts of size‐ and age‐related changes in energetic allocation on population‐wide recruitment and the factors leading to sexual transition at the individual level of NS *C. striata* are unknown, but likely include size, age, energetic status, densities of males and mating system (*e.g*., harem, lek, aggregation) all of which may vary across a distribution. Although the authors did collect a few individuals that were in transition, they did not explore energetics in these fish because of small sample sizes and because energetics of transition were not a focus of this study. Further investigation on regional differences in size and age at transition, mating systems and sex ratios is needed.

A notable trend in energetic allocation of NS *C. striata* throughout their distribution was the significantly lower somatic and reproductive energetic values in MA fish. There are several possible explanations for this result. To start, and as mentioned above, NS *C. striata* migrating to the northern portion of their distribution have a longer migration and appear to arrive in lower energy status that cannot be recovered before spawning. MA fish also have a shorter spawning season and to match the cumulative energy output as seen in DE fish, they would need to double their daily GTE output (assuming similar spawning frequency). Next, larger body sizes are advantageous for longer migrations for both swimming efficiency and capacity to store energy (Slotte, 1999). NS *C. striata* exhibit some site fidelity (Moser & Shepherd, 2008), which suggests MA fish likely return to the same spawning region the following year. Therefore, NS *C. striata* in MA would benefit by allocating some energy towards somatic growth during the summer, which could be at the cost of gonad development. Moreover, NS *C. striata* biomass has been increasing in the northern regions leading to higher population density in the MA region. Density dependence is known to affect growth and fecundity for a number of fish including the round goby (*Neogobius melanostomus*; Gutowsky & Fox, 2012; Houston *et al*., 2014), largemouth bass (*Micropterus salmoides*; Pritt *et al*., 2020), and European anchovy (*Engraulis encrasicolus*; Basilone *et al*., 2020). High population density in the MA region could therefore negatively affect energy allocation and reproductive output. Finally, there could be dietary differences throughout the range as has been seen in NS *C. striata* from Southern New England and northward (McMahan *et al*., 2020), affecting energetic intake and allocation. Altogether the low energetic status of MA fish could be because of a combination of abiotic and biotic factors.

Although there were intraspecific differences across the distribution of NS *C. striata*, altogether their energy allocation patterns were suitable for spawning at higher latitudes where productivity is pulsed. Specifically, NS *C. striata* used both endogenous and exogenous energy sources during spawning and maintained high gonad energetic output, as compared to fish with protracted spawning seasons (Alonso‐Fernández & Saborido‐Rey, 2012). Spawning strategies and energy allocation in NS *C. striata* may differ from other stocks, the SES and Gulf of Mexico (GOMexS) stocks, because of differences in location and population dynamics. SES *C. striata* are non‐migratory (Watanabe, 2011) and spawn from March to May with potentially a secondary spawning period in September to October (Wenner *et al*., 1986). Notably, SES *C. striata* have lower GSI than NS *C. striata* (*c*. 2–3; Link, 1980 *vs. c*. 6–8 Slesinger *et al*., 2021). Across the distribution of the SE stock, variation within the population also exists for size at age in males and females (McGovern *et al*., 2002). The GOMex stocks are also non‐migratory and spawn from December to April (Hood *et al*. 1994). GOMexS *C. striata* grow faster and have shorter lifespans than SES and NS *C. striata* (Hood *et al*. 1994). Between the three stocks, NS *C. striata* are the only fish to seasonally migrate, which may require differing energy allocation strategies before and postspawning. To the best of the authors' knowledge, there are no studies focused on the dynamics of energy allocation throughout a spawning season of SES and GOMexS *C. striata*. Nonetheless, based on previous studies, the authors would speculate SES and GOMexS *C. striata* are predominantly income breeders and energetic analyses would reflect maintained somatic energy stores throughout spawning. Across their entire range, reproductive plasticity of *C. striata* may help explain how a tropical serranid has successfully colonized temperate waters when many other serranids are restricted to subtropical and tropical waters. Future studies on the energetic allocation in *C. striata* throughout the entire range would provide information important for the species as a whole and add context to the results in this study.

NS *C. striata* collections were opportunistic and study limitations (discussed in more detail in Slesinger *et al*., 2021) led to sampling across 2 years, where NJ and MA were sampled in 2018 and in generally shallower water, whereas DE and VA were sampled in 2019 in deeper and more offshore water. Across space, the authors chose to evaluate energetics across the range of samples that were clustered by the states of the U.S.A. because they set their own harvest limits (size, season and catch) and to increase sample sizes (by spatial unit) for statistical analyses. It should be noted that the average latitudinal distance between sampling sites was similar (*c*. 1.4–1.6°). An improved spatial sampling design would be time intensive and expensive, but could fill important data gaps between the sampling locations and address differences within sites (*i.e*., depth). Across time, ocean conditions were similar between the 2 years and the timing of peak spawning corroborated with other studies of NS *C. striata*, but interannual differences between NS *C. striata* energetics and reproductive output could still have occurred. For the northern regions, the trend of lower energetics from south to north is reflected in a comparison between NJ and MA (*i.e*., see Table 5). Substantial variation in estimated annual fecundity has been documented for SES *C. striata* (Klibansky & Scharf, 2017), suggesting the species has considerable flexibility in annual energy allocation to reproduction. Time‐series of fecundity and energetics data are needed to fully understand the drivers and pathways regulating reproductive potential. Nonetheless, these data provide a snapshot into potential energetic conditions NS *C. striata* can experience throughout their distribution and may serve as a window into interannual differences seen in NS *C. striata* recruitment. For example, years with strong cohorts of NS *C. striata* can arise from spatially heterogeneous production, such as the 2011 year class for which a majority of age‐0 fish were from north of the Hudson Canyon, and could also have been driven by warm winter conditions of 2012 (Miller *et al*., 2016). Therefore, recruitment success can occur unevenly throughout the distribution, and the data of this study provide evidence that there are major differences in adult energetics and reproductive potential, which may be a driver.

In addition, although this would not affect the current interpretation of the data, because of time constraints, the authors did not dissect nor measure the energetics of the viscera, another potential storage depot for *C. striata*, particularly as other energy storage sites reach capacity (Wuenschel *et al*., 2013). Although the authors may have overlooked a potential energy source that could differ regionally, they found similar trends in liver, muscle and gonad across the regions. Differences in viscera energy could explain higher gonad energetics in some of the fish; nonetheless, this information would likely be additive to liver energetics as fish with higher gonad energy typically had higher liver energy.

Overall, the authors found regional differences in NS *C. striata* energy allocation associated with differences seen in reproductive energy investment and output. The data of this study suggest that NS *C. striata* closer to the centre of their geographic range had higher energy allocation towards spawning than those from more northern locations where abundances have increased recently. Although not directly tested in this study, there are multiple sources of regional differences that can give rise to the energetic dissimilarities including abiotic factors (*i.e*., temperature), migration distance, diet and density dependence. That these regional drivers may affect energy allocation towards reproduction is important as NS *C. striata* are managed as one stock with regional quota differences apportioned to individual states by biomass. As ocean warming continues along the U.S. Northeast Shelf (Chen *et al*., 2020), and NS *C. striata* centre of biomass expands northward (Kleisner *et al*., 2017), it is important to address the interaction between shifting biomass, where a higher proportion of fish are spawning further north along the shelf, and potential energetic limitations or benefits at that location. Ocean warming can occur at different rates throughout seasons, and the view of longer migration distances as a result of summer warming assumes a constant winter temperature. Should there be increased winter warming opening available habitat further inshore and north, the final migration distance may be reduced, or lead to continued separation of the species which usually mix during the winter. Finally, this research is focused on a single species of fish but has implications for other species with wide latitudinal distributions and/or prespawning migrations; differences throughout the distribution of NS *C. striata* led to substantial dissimilarities in energetic allocation and reproduction, relevant for future management, especially under continued ocean warming.

## AUTHOR CONTRIBUTIONS

E.S.: ideas, data generation, data analysis and manuscript preparation. K.B.: data generation, data analysis and manuscript preparation. M.W.: data analysis and manuscript preparation. G.K.S: data analysis, manuscript preparation and funding.

## Supporting information


**Appendix** **S1** Supporting information.
