# Supplementary material for: Regional differences in energy allocation of black sea bass ( Centropristis striata ) along the U.S. Northeast Shelf (36°N to 42°N) and throughout the spawning season
Source: J Fish Biol. 2022 Mar 9;100(4):918–34. doi: 10.1111/jfb.15023 (PMC9310597; doi:10.1111/jfb.15023)
Supplement: Supplementary file 1 — Appendix S1 Supporting information. [file JFB-100-918-s001.docx]

**SUPPLEMENTAL INFORMATION**

**Regional differences in energy allocation of black sea bass (*Centropristis striata*) along the US Northeast Shelf (36°N - 42°N) and throughout the spawning season**

Emily Slesinger^1*^, Kiernan Bates^1^, Mark Wuenschel^2^, and Grace K. Saba^1^

TABLES

SI Table 1. The models used in analysis, their distribution family and link functions.

| Analysis | Response variable | Model | Distribution family | Link |
| --- | --- | --- | --- | --- |
| Compositional | LC (g lipid/g dry weight) | GLM | Beta | Logit |
|  | ED (kJ/g wet weight) | GLM | Gamma | Inverse |
|  | TE (kJ) | GLM | Gamma | Log |
| Seasonal | LTE | GAM | Gamma | Log |
|  | GTE | GAM | Gamma | Log |

SI Table 2. BIC values used to compete the null and regional models. Bold values indicate the lowest BIC and the chosen model

| Tissue | Sex | Model | Measurement | | |
| --- | --- | --- | --- | --- | --- |
|  |  |  | LC (g/g DW) | ED (kJ/g WW) | TE (kJ) |
| Muscle | All | Null | **-1554.69** | 262.97 | NA |
|  |  | Regional | -1540.56 | **215.11** | NA |
| Liver | F | Null | -225.20 | 652.30 | 1376.39 |
|  |  | Regional | **-237.98** | **646.75** | **1364.76** |
|  | M | Null | -195.60 | **446.64** | 1006.90 |
|  |  | Regional | **-198.83** | 449.71 | **1004.27** |
| Gonad | F | Null | -549.70 | 485.49 | 1642.10 |
|  |  | Regional | **-566.83** | **478.55** | **1628.76** |
|  | M | Null | -518.79 | **129.61** | **996.12** |
|  |  | Regional | **-522.34** | 141.43 | 999.10 |

FIGURES


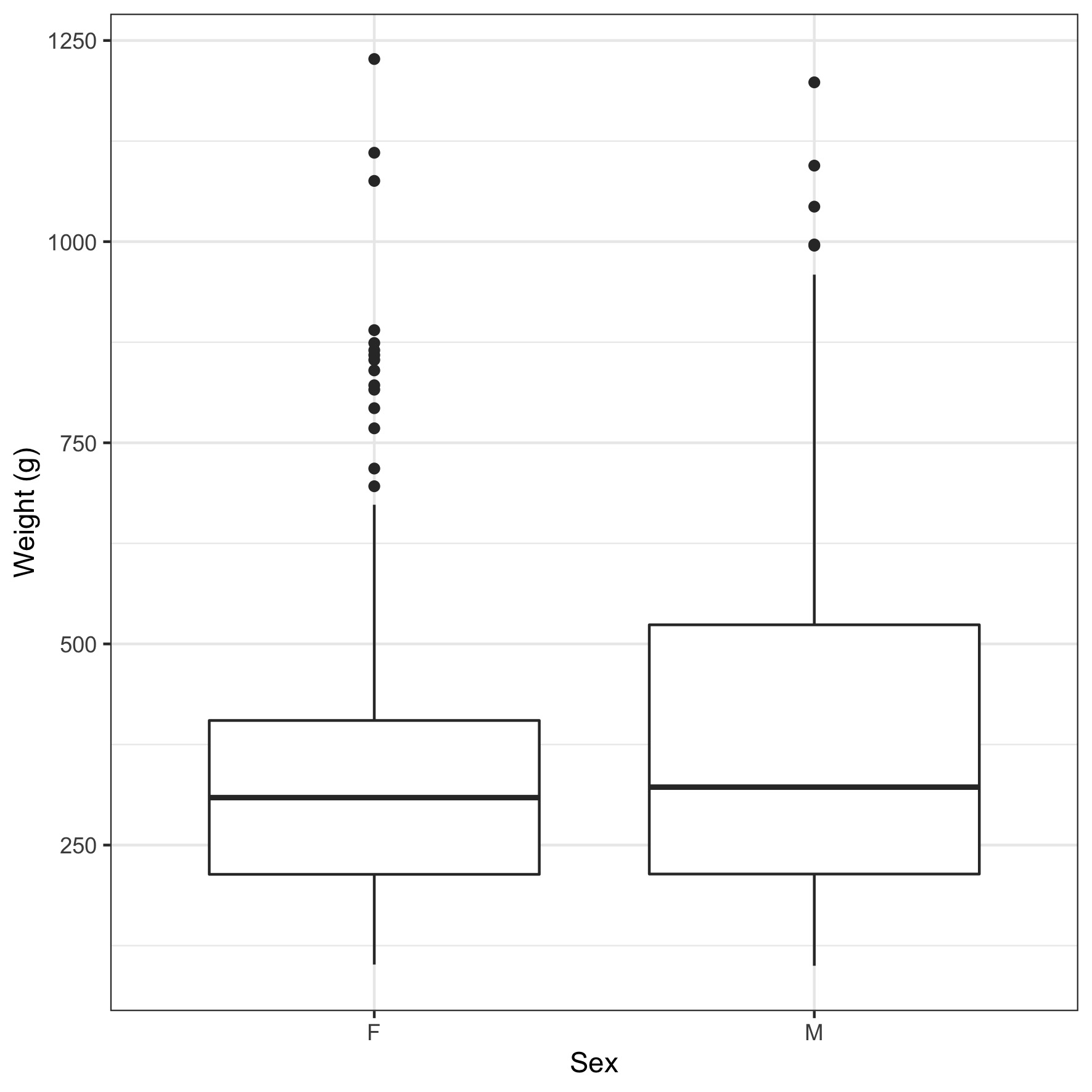


SI Figure 1. The size distribution between female and male fish of individuals used in this study.


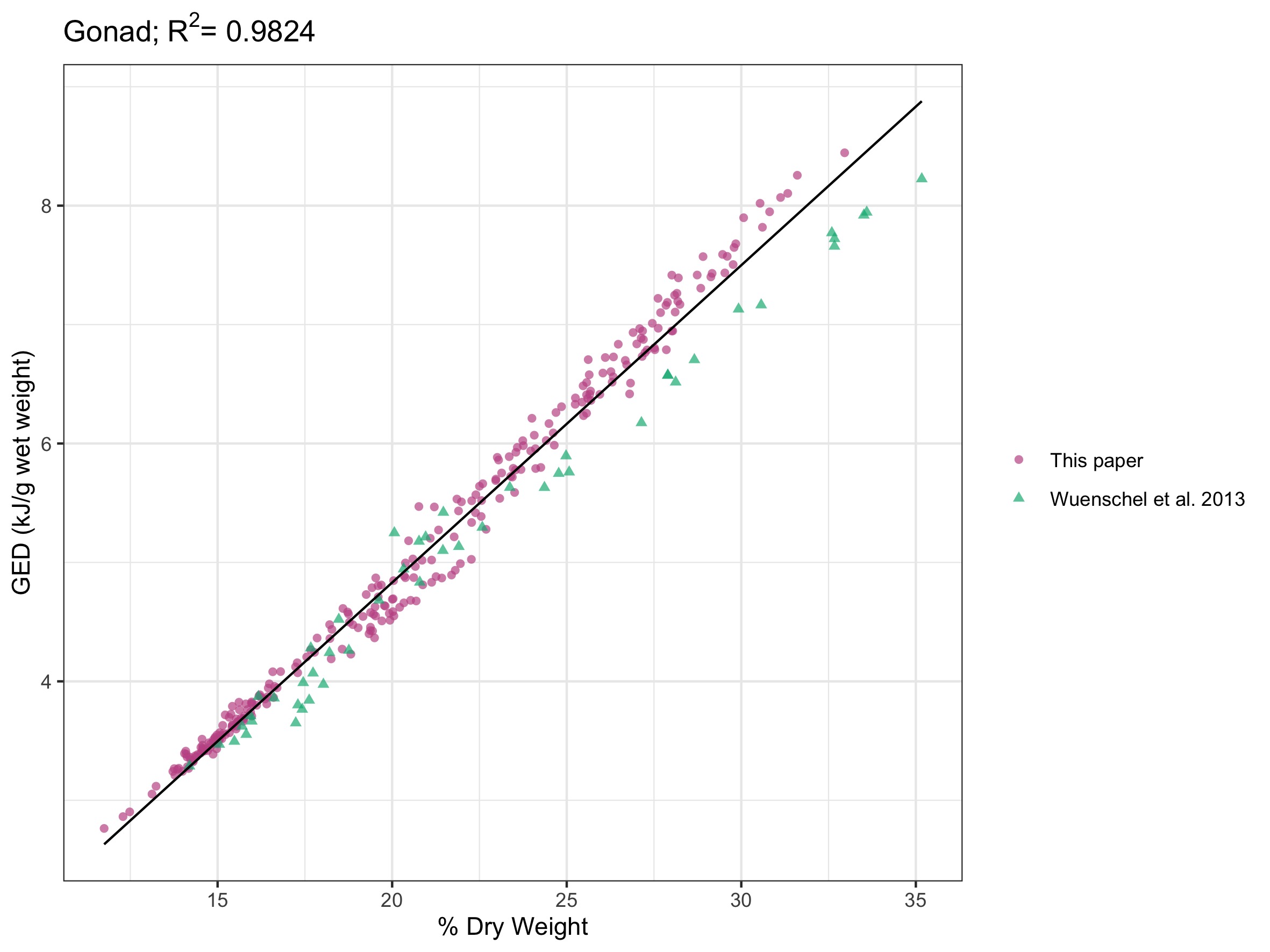


SI Figure 2. Linear trend of gonad % dry weight to GED for this paper and data from Wuenschel et al. 2013 to assess fit of our estimated ED values from those already published for *C. striata*.


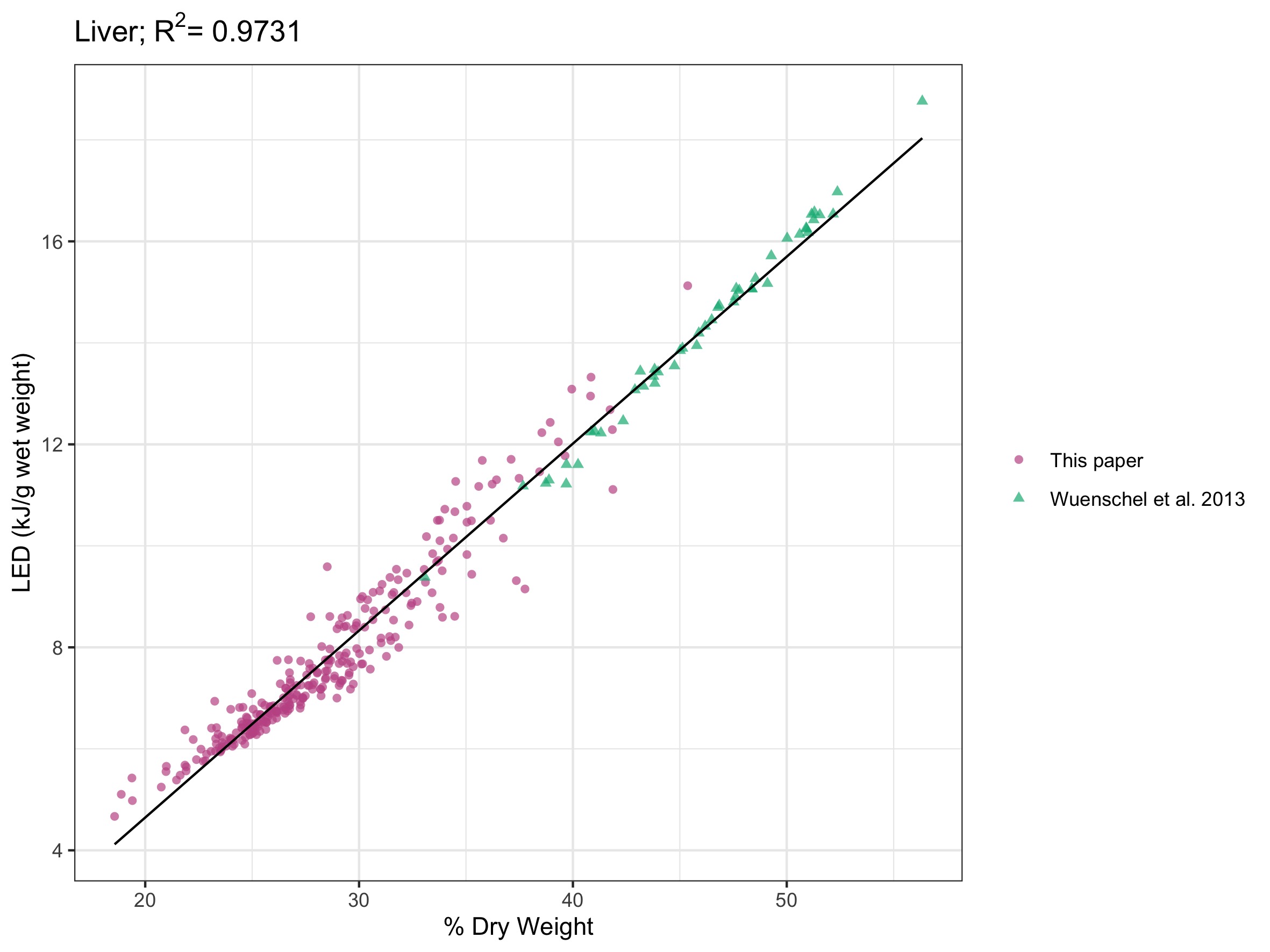


SI Figure 3. Linear trend of liver % dry weight to LED for this paper and data from Wuenschel et al. 2013 to assess fit of our estimated ED values from those already published for *C. striata*.


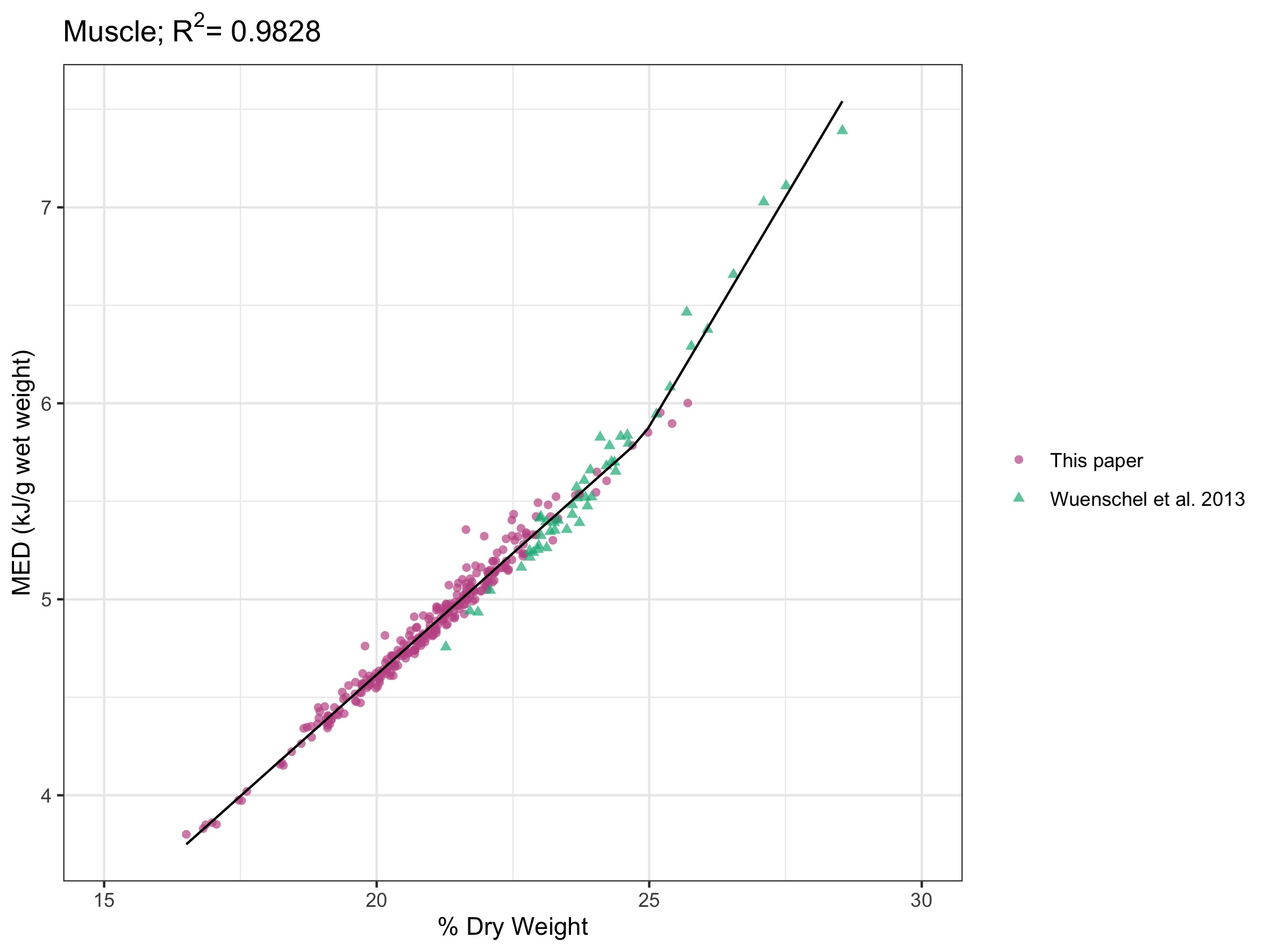


SI Figure 4. Regression of muscle % dry weight to MED, using a broken stick regression, for this paper and data from Wuenschel et al. 2013 to assess fit of our estimated ED values from those already published for *C. striata*.
